# Examining the role of knowledge and trust on vaccine confidence in North Dakota *among university students, faculty, and staff*

**DOI:** 10.1186/s12889-024-19056-x

**Published:** 2024-06-07

**Authors:** Mary Bruns, Tanis Walch, Claire Wagner, Rylee Bergeron, Soojung Kim

**Affiliations:** 1 School of Medicine and Health Sciences, University of North Dakota, 1301 North Columbia Road Stop 9037, Grand Forks, ND 58202 USA; 2 Department of Education, Health & Behavior, University of North Dakota, 231 Centennial Drive Stop 7189, Grand Forks, ND 58202 USA; 3 Department of Communication, University of North Dakota, 221 Centennial Dr. Stop 7169, Grand Forks, ND 58202-7169 USA

**Keywords:** Vaccine confidence, Vaccine knowledge, Trust, Media, Rural

## Abstract

**Background:**

Vaccination is one of the greatest tools for individuals to stay healthy. Individuals are, however, often exposed to misinformation via digital and social media, and thus, may miss the opportunity to develop scientific knowledge about vaccines and trust in relevant stakeholders. This has a damaging impact on vaccine confidence. Understanding vaccine confidence is particularly important in North Dakota, where vaccination rates are lower than national averages.

**Objectives:**

The objectives of this research are to examine the association between vaccine confidence and three potential sources of it, namely, trust, vaccine knowledge, and vaccine information sources and to investigate the relative strength of three vaccine confidence sources, while accounting for covariates.

**Methods:**

Students (*n* = 517, 56.6%) and staff and faculty (*n* = 397, 43.4%) at the University of North Dakota (*n* = 914) completed an online survey. Logistic regressions estimated odds ratios (OR) and 95% confidence intervals (CI) for associations among trust in doctors, family/friends, government health agencies, charitable organizations, and religious organizations, vaccine knowledge, vaccine information sources as well as vaccine confidence, accounting for gender, race, marital status, age, religion, political ideology, education, and health status.

**Results:**

The mean age of participants was 29.43 years (SD = 13.48). Most were females (71.6%) and white (91.5%). Great trust in doctors (OR = 3.29, *p* < 0.001, 95%CI 1.89, 5.73) government health agencies (OR = 2.95, *p* < 0.001, 95%CI 2.13, 4.08) and vaccine knowledge (OR = 1.28, *p* < 0.001, 95%CI 1.18, 1.38) had higher odds of vaccine confidence. Using Internet Government source as the primary source of vaccine information (OR = 1.73, *p* < 0.05, 95%CI 1.22, 2.44) showed higher odds of vaccine confidence before all independent variables were introduced, but it became non-significant after they were introduced. Trust in government health agencies showed strongest associations with vaccine confidence.

**Conclusion:**

Multiple stakeholders are necessary to ensure verified, accessible, and accurate information in order to advance vaccine confidence in rural, conservative areas.

Vaccinations annually prevent between 3.5 and 5 million deaths [1], as routine vaccination is an important primary prevention effort in public health. Despite these successes, we face a high level of vaccine hesitancy, especially after the COVID-19 pandemic [2]. Vaccine hesitancy is defined as the delay in acceptance or refusal of vaccines despite availability of vaccination services. Vaccine hesitancy is complex and context specific varying across time, place, and vaccines. It includes factors such as complacency, convenience, and confidence [3]. Vaccine hesitancy worldwide is fueled by a myriad of factors, including religious and historical negative experiences with foreign healthcare, adverse effects of vaccination in middle- and low-income countries, and concerns of vaccine safety in high-income countries [4]. In America, vaccine hesitancy is an especially cumbersome issue in rural and generally conservative-leaning states [5]. For instance, according to the North Dakota Department of Health and Human Services, only 21.4% of adults aged 18–49 years had received an influenza vaccine compared to the national average of 37.1% [6, 7].

Previous research has identified several factors contributing to a recent surge in vaccine hesitancy [8], including political and religious ideologies [9], media misinformation [10], family/friend influences [11], and socioeconomic status [12]. The process of developing vaccine hesitancy appears to start from individuals’ exposure to different information sources. Individuals’ high and low reliance on social media and academic sources, respectively, resulted in their vulnerability to misinformation or “fake news” [13, 14]. Misinformation especially as a result of uncertainty during the COVID-19 pandemic, has been a common cause contributing to decreased vaccine acceptance [15, 16].

Detrimental effects of exposure to vaccine-related misinformation could be mitigated by stronger trust in stakeholders related to vaccinations, such as healthcare providers, science experts, and government agencies [17]. Addressing vaccine hesitancy and rebuilding vaccine confidence has, thus, become a formidable issue for the public health and healthcare realm. The CDC defines vaccine confidence as “the belief that the vaccine works, that it is safe, and that it is part of a trustworthy medical system” [18]. Vaccine confidence is a multi-dimensional construct, including scientific knowledge, trust in authority, risk perception, media use, as well as socio-demographic characteristics, including political and religious ideologies [19]–[24]. This reiterates the fact that trust plays an integral role in addressing vaccine hesitancy and building vaccine confidence encompassing vaccine products, providers, and stakeholders creating and enforcing vaccine policies [25]. In attempting to re-build trust and encourage vaccine confidence, an essential part is through the provisions of trustworthy information sources to aid in increasing vaccine knowledge [26].

The University of North Dakota, with the support of the North Dakota Department of Health and Human Services, plans to launch a campus-wide campaign named Vax Venture to increase vaccine confidence among all routine vaccinations. Addressing the gap in the literature concerning the relationships among the use of vaccine information sources, vaccine knowledge, and trust contributing to vaccine confidence, the objectives of this research are twofold: (1) to examine the association between vaccine confidence and three potential sources of it, namely, trust, vaccine knowledge, and vaccine information sources, while accounting for demographics, socioeconomic status, and health characteristics; and (2) to investigate the relative strength of three vaccine confidence sources, after adjusting for covariates. The findings of this study would contribute to implementing effective public health programs that appropriately allocate resources toward re-instating trust, increasing confidence, and decreasing preventable disease.

## Methods

An online cross-sectional survey was conducted using Qualtrics. This research was approved by the IRB at the University of North Dakota (UND), which considered the study exempt (IRB # IRB005352).

### Sample

Students, staffs, and faculty over the age of 18 at UND were invited to participate in the survey. The enrollment began in October 2022, the last participant answered in November 2022, and the online survey was closed in December 2022. In total, 1,043 participants accessed the survey, among them, 1,038 were eligible, indicating that they were an on-campus student, faculty or staff member at UND. We observed a response rate of 7.90% (1,038/13,143) among eligible individuals invited to participate. Non-responses may be due to various factors including time restraints or disinterest [27, 28]. Distance students and remote employees were not eligible. In total, 914 participants completed the survey and were used in the final data analysis.

### Procedure

All students, faculty, and staff at UND were recruited via mass email, which contained a link to access the Qualtrics survey. Those who access the survey link were first asked to read and agree to a consent form. All participants who consented and reported that they were a student, faculty, or staff at UND were eligible to proceed. Participants were then asked to answer demographic questions before answering questions regarding their vaccination status, beliefs, and attitudes about vaccination regarding the following routine vaccines: COVID-19, influenza, HPV, MenB, MenACWY, Tdap, MMR, varicella, shingles, Hepatitis A, and Hepatitis B. The survey took approximately 15 min. After completing the survey, participants received a $10 gift card in exchange for their participation in the survey.

### Measures

The survey questionnaire included questions measuring participants’ trust in relevant stakeholders, vaccine knowledge, information sources, vaccine confidence, and sociodemographic and health characteristics. Questions included multiple response options adapted by scales used in previous literature and previously piloted iterations of survey questionnaire [29]–[36]. The reliability statistics for constructed variables were found to be satisfactory (see the Variable Construction section below). The pilot-testing of the survey questionnaire among a small group of undergraduate students demonstrated that there was little to no confusion or questions regarding the questionnaire.

### Outcome (vaccine confidence)

*Vaccine confidence*.

Confidence in vaccine information sources was measured by asking the following question: “Overall, how confident are you that you could get advice or information about vaccines if you needed it?” Reponses ranged from 1 (“*not at all*”) to 5 (“*a lot*”) [35].

### Independent variables (Trust, Knowledge, and information source)

*Trust.* Trust in vaccine information sources was measured by asking the following question: “In general, how much would you trust information about vaccines from each of the following?” Five sources of trust were provided: (1) “A doctor” (2), “Family or friends” (3), “Government health agencies” (4), “Charitable organizations” (5), “Religious organizations and leaders” [29, 35]. Response options for each source of trust ranged from 1 (“*not at all*”) to 4 (“*a lot*”).

#### Vaccine knowledge

Questions seeking individual knowledge of vaccines and the diseases they prevent included seven true-or-false questions about the influenza (flu) vaccine (e.g., “The influenza vaccine is only minimally effective”) [31, 32], six true-or-false questions about the HPV vaccine (e.g., “Men cannot get HPV”) [33], three true-or-false questions about the Tdap vaccine (e.g., “Adults should get a tetanus booster every 10 years”) [30], and three true-or-false questions about the shingles vaccine (e.g., “The chance of developing shingles increases with age”). Three response options were provided for each question: “True”, “False”, or “I don’t know” [34]. Detailed information about the creation of vaccine knowledge variable is presented under the results section below.

#### Information sources

To measure individuals’ *general* information sources in terms of vaccine information, the following question was asked: “Imagine that you had a strong need to get information about vaccines. Where would you go first?” [35]. Response options included (1) “Books” (2), “Brochures, pamphlets, etc.” (3), “Vaccine organizations” (4), “Family” (5), “Friend/Co-worker” (6), “Doctor or health care provider” (7), “Internet” (8), “Library” (9), “Magazines” (10), “Newspapers” (11), “Telephone information number” (12), “Complementary, alternative, or unconventional practitioner” (13), “Other (please specify).

Another question was asked to identify individuals’ *Internet* sources regarding vaccine information: “If you search the Internet for vaccine information, please rank the following from 1 to 12, with 1 being your first place to look for information. Twelve Internet sources included: (1) “Blogs” (2), “Facebook” (3), “Twitter” (4), “Instagram” (5), “Tik Tok” (6), “YouTube” (7), “TV news website (NBC, FOX, CNN, etc.)” (8), “Newspaper/magazine website (Wall Street Journal, The Atlantic, etc.)” (9), “Government websites that end in .gov” (10), “Websites of nonprofits that end in .org” (11), “Websites of universities that end in .edu”, and (12) “Other online sources”. Detailed information about the creation of information source variables is presented under the results section below.

### Covariates

Covariates regarding demographic characteristics and socioeconomic status included [35]: gender, race (white vs. non-white), political ideology (liberals, neutral, vs. non-liberals), education level (some college or more vs. lower), marital status (married vs. single), general health (good vs. poor), religious ideology (atheist vs. non-atheist), age, and insurance status (insured vs. not insured) [36]. Original questions had multiple response options, for example, race included “White”, “American Indian/Alaska Native”, “Black or African American”, “Asian or Pacific Islander”, “Hispanic, Latinx or Spanish”, “Mexican, Mexican American, Chicano”, “Puerto Rican”, “Cuban”, “Another Hispanic, Latinx, or Spanish origin” but were merged into top responses.

### Variable construction

For the vaccine knowledge variable, responses were recoded depending on the correct answer for a question. For all questions “I don’t know” was coded as “incorrect”. For each set of knowledge questions (i.e., flu, shingles, tetanus, and HPV), “correct” answers were totaled to create a knowledge index for each set. Once all correct answers between all sets of knowledge questions were totaled, a final knowledge index was created to determine the level of vaccine knowledge. Higher index scores indicated higher knowledge.

For information source variables, the top ranked answers from respondents were used to create three dichotomous variables. First, 55.5% of respondents selected “doctor or health care provider” (out of 13 response options) as their answer to the following question – “Imagine that you had a strong need to get information about vaccines. Where would you go first?”, which led to the creation of expert primary source variable (yes/no). Second, 32.8% of respondents selected “Internet” (out of 13 response options) as their answer to the aforementioned question, which led to the creation of Internet primary source variable (yes/no). Third, 49.3% of respondents selected “Government websites that end in .gov” (out of 12 response options) as their answer to the following question – “If you search the Internet for vaccine information, please rank the following from 1 to 12, with 1 being your first place to look for information.” This led to the construction of government primary source variable (yes/no). These three information source variables are reflective of popular vaccine information sources available to the general public [13, 37].

### Data analyses

First, descriptive statistics were obtained. Next, logistic regressions estimated regression coefficients (B) and 95% confident intervals (CI) for associations between independent variables – trust in five sources: (1) doctors (2), family/friends (3), government health agencies (4), charitable organizations, and (5) religious organizations, vaccine knowledge and three information sources: (1) expert primary source (2), Internet primary source, and (3) government primary source – and the dependent variable, vaccine confidence, accounting for gender, race, political ideology, education level, marital status, self-reported health status, religion, age, and insurance status. Given the low number of missing variables, a complete-case analysis was deemed appropriate. As such, missing and incomplete responses were excluded.

## Results

### Participant characteristics

Table 1 presents participants’ demographic characteristics. The mean age of participants was 29.43 (SD = 13.48). Most were female (*n* = 634, 71.6%) and white (*n* = 827, 91.5%). Most participants identified as liberal (*n* = 410, 45.5%), high general health (*n* = 510, 57.4), catholic or protestant (*n* = 538, 59.8%), student (*n* = 517, 0.6%), and single (*n* = 489, 54.1%).

**Table 1:** Participant Demographic Characteristics

Characteristics	*N*	%
**Sex**		
Female	634	71.6
Male	251	28.4
Total	885	100
**Race/ethnicity**		
White	827	91.5
Non-White	77	8.5
Total	904	100
**Education**		
≤ Some college	472	52.9
≥ 4-year degree	421	47.1
Total	893	100
**Household income**		
<$50,000	278	37.7
≥$50,000	459	62.3
Total	737	100
**Religion**		
Catholic/Protestant	538	59.8
All other religions	362	40.2
Total	900	100
**Political ideology**		
Liberal	410	45.5
Neutral	225	25.0
Conservative	266	29.5
Total	901	100
**Health status**		
High	510	57.4
Low	379	42.6
Total	889	100
**Insurance status**		
Insurance	863	97.5
No insurance	22	2.5
Total	885	100
**Marital status**		
Married/separated/divorced	415	45.9
Single	489	54.1
Total	904	100
**Age**		
≤30	543	65.6
> 31	285	34.4
Total	828	100
**Job**		
Student	517	56.6
Faculty	97	10.6
Staff	300	32.8
Total	914	100
**Vaccination status—received vaccine**		
COVID-19 Flu	154 557	19.5 62.8
HPV	443	50.0

Table 2 presents trust, knowledge, and information source among participants. The majority of respondents (77.1%) reported high levels of trust in doctors, while trust in family/friends (6.1%), government health agencies (26.7%), charitable organizations (6.3%), and religious organizations (1.5%) reported lower levels of trusting these sources “a lot”. The overall mean knowledge score was 9.85 (SD = 3.33). Most respondents used an expert source (55.5%) and government internet sources (50.7%) for their first source when searching for vaccine information while broadly searching the internet was less common (32.8%). The majority of participants (62.8%) had received the flu vaccination, and half (50.0%) have received the HPV vaccination.

**Table 2:** Descriptive characteristics of trust, knowledge, and primary information source

**2.1 Trust n (%)**							
	Trust in doctor	Trust in family/friends		Trust in government health agencies	Trust in charitable organizations		Trust in religious organizations
Not at all	2 (0.4)	55 (11.6)		41 (8.7)	93 (19.7)		274 (57.9)
A little	13 (2.7)	203 (42.9)		83 (17.6)	186 (39.3)		139 (29.4)
Some	94 (19.9)	186 (39.3)		222 (47.0)	164 (34.7)		53 (11.2)
A lot	364 (77.0)	29 (6.1)		126 (26.7)	30 (6.3)		7 (1.5)
**2.2 Knowledge score**							
	M	SD		Min	Max		N
Flu	4.68	1.46		0	6		466
HPV	2.86	2.05		0	6		463
Tdap	2.27	0.71		0	3		222
Shingles	2.27	1.07		0	3		461
All	9.85	3.33		0	15		458
**2.3 Primary information source n (%)**							
	Expert primary source		Internet primary source			Gov primary source	
First choice	262 (55.5)		155 (32.8)			229 (50.7)	
Other choices	210 (44.5)		317 (67.2)			223 (49.3)	

### Hypothesis 1

The first hypothesis predicted that there would be an association between vaccine confidence and three sources of it, namely, trust, vaccine knowledge, and vaccine information sources, while accounting for demographics, socioeconomic status, and health characteristics. This hypothesis aims to estimate an association between each source and vaccine confidence.

#### Main results

In the fully adjusted model (Model 4) where all independent variables were included (see Table 3), trust in doctors/health care workers (OR = 1.91, *p* < 0.001, 95%CI 1.41, 2.59), trust in government health agencies (OR = 2.72, *p* < 0.001, 95%CI 2.01, 3.67), and vaccine knowledge (OR = 2.26, *p* < 0.001, 95%CI 1.75, 2.92) were associated with vaccine confidence significantly and positively, after accounting for covariates. This indicates that those who have higher trust in doctors/healthcare workers, government health agencies, and vaccine knowledge have increased odds of vaccine confidence.

**Table 3:** Logistic regression analysis factors contributing to vaccine confidence (Models 1 & 2)

		Model 1						Model 2					
		Crude			Adjusted			Crude			Adjusted		
		Beta	OR	95% CI	Beta	OR	95% CI	Beta	OR	95%CI	Beta	OR	95% CI
Trust in doctor		0.70	2.01*	(1.58, 2.55)	0.72	2.05*	(1.56, 2.69)						
Trust in family/friend		-0.17	0.84	(0.70, 1.01)	-0.18	0.84	(0.68, 1.03)						
Trust in government health agencies		1.15	3.14*	(2.52, 3.92)	0.99	2.68*	(2.06, 3.49)						
Trust in charitable organizations		0.18	1.19	(0.99, 1.44)	0.08	1.08	(0.87, 1.34)						
Trust in religious organizations		-0.18	0.83	(0.69, 1.00)	0.05	1.05	(0.84, 1.30)						
Knowledge								0.82	2.27*	(1.93, 2.68)	0.80	2.22*	(1.78, 2.75)

*p < 0.001

Covariates: gender, race (white vs. others), political ideology (liberal vs. others), higher education level, marital status (single vs. others), general health (good vs. poor), religious ideology (atheist vs. others), age, and insurance status (insured vs. not insured). Referent categories for information source were all other sources selected as the first source of vaccine information

OR = odds ratio; CI = confidence interval

#### Supplementary results

Models 1, 2, and 3 in Tables 4 and 3 demonstrate an individual association between trust variables and vaccine confidence, between vaccine knowledge and vaccine confidence, and between vaccine information sources and vaccine confidence, respectively. First, as shown in Model 1 in Table 4, after adjusting for covariates, trust in doctors (OR = 2.05, *p* < 0.001, 95%CI 1.56, 2.69), and trust in government health agencies (OR = 2.68, *p* < 0.001 95% CI 2.06, 3.49) showed higher odds of vaccine confidence.

**Table 4:** Logistic regression analysis factors contributing to vaccine confidence (Models 3 & 4)

	Model 3					Model 4			
	Crude			Adjusted			Adjusted		
	Beta	OR	95% CI	Beta	OR	95% CI	Beta	OR	95% CI
Trust in doctor							0.65	1.91*	(1.41, 2.59)
Trust in family/friends							-0.19	0.83	(0.66, 1.03)
Trust in government health agencies							1.00	2.72*	(2.01, 3.67)
Trust in charitable organizations							0.09	1.09	(0.86, 1.38)
Trust in religious organizations							0.01	1.01	(0.80, 1.28)
Knowledge							0.82	2.26*	(1.75, 2.92)
Expert primary source	-0.14	0.87	(0.56, 1.35)	0.09	1.10	(0.64, 1.87)	0.19	1.20	(0.62, 2.33)
Internet primary source	-0.05	0.96	(0.60, 1.52)	-0.15	0.86	(0.50, 1.50)	-0.21	0.81	(0.41, 1.59)
Government primary source	0.90	2.45*	(1.84, 3.26)	0.55	1.73*	(1.22, 2.44)	0.65	1.91*	(1.41, 2.59)

*p < 0.001; Covariates: gender, race (white vs. others), political ideology (liberal vs. others), higher education level, marital status (single vs. others), general health (good vs. poor), religious ideology (atheist vs. others), age, and insurance status (insured vs. not insured). Referent categories for information source were all other sources selected as the first source of vaccine information

OR = odds ratio; CI = confidence interval

Expert primary source: Doctor/health care worker as the first source of information (vs. other choices [ranks 2–13])

Internet primary source: Internet as the first source of information (vs. other choices [ranks 2–13])

Government primary source: Government websites as the first Internet source for information (vs. other choices [ranks 2–13])

Second, vaccine knowledge showed higher odds of vaccine confidence both before (OR = 2.27, *p* < 0.001, 95%CI 1.93, 2.68) and after adjusting for covariates (OR = 2.22, *p* < 0.001, 95%CI 1.78, 2.75) (see Model 2 in Table 4), suggesting that individuals who have higher levels of knowledge tend to have higher vaccine confidence. Third, the government primary source variable had higher odds of vaccine confidence both before (OR = 2.45, *p* < 0.001, 95%CI 1.84, 3.26) and after adjusting for covariates (OR = 1.73, *p* < 0.001, 95%CI 1.22, 2.44) (see Model 3 in Table 3). This indicates that higher levels of confidence are observed in individuals who use a government source as their initial destination for vaccine information.

When Model 4 was compared to adjusted Model 1, trust in doctors showed a 6.83% decrease, and trust in government health agencies showed a 1.49% increase in odds ratios. Compared to adjusted Model 2, vaccine knowledge showed a 1.80% increase in odds ratio. Compared to adjusted Model 3, the government primary source variable showed 36.42% decrease in odds ratio, not being associated with vaccine confidence anymore.

### Hypothesis 2

The second hypothesis predicted the relative strength of vaccine confidence sources, after adjusting for covariates. This hypothesis investigates the comparative strength of sources contributing to vaccine confidence collectively.

#### Main results

In the fully adjusted model (see Model 7 in Table 5),as trust in doctors (OR = 1.91, *p* < 0.001, 95%CI 1.41, 2.59), trust in government health agencies (OR = 2.72, *p* < 0.001, 95%CI 2.01, 3.67), and vaccine knowledge (OR = 2.26, *p* < 0.001, 95%CI 1.75, 2.92) increased by one unit among a standardized scale, odds of vaccine confidence increased by 91%, 172%, and 126%, respectively. These findings indicate that individuals who trust in government agencies have highest rates of vaccine confidence, followed by vaccine knowledge and trust in doctors.

**Table 5:** Multiple regression analysis factors contributing to vaccine confidence (Models 5, 6, & 7)

	Model 5			Model 6				Model 7			
	Beta	OR	95% CI		Beta	OR	95% CI		Beta	OR	95% CI
Trust in doctor	0.72	2.06*	(1.56, 2.69)		0.64	1.90*	(1.43, 2.52)		0.65	1.91*	(1.41, 2.59)
Trust in family/friends	-0.18	0.84	(0.68, 1.03)		-0.20	0.82	(0.66, 1.01)		-0.19	0.83	(0.66, 1.03)
Trust in government health agencies	0.99	2.68*	(2.06, 3.49)		0.99	2.69*	(2.04, 3.53)		1.00	2.72*	(2.01, 3.67)
Trust in charitable organizations	0.76	1.08	(0.87, 1.34)		0.07	1.08	(0.85, 1.35)		0.09	1.09	(0.86, 1.38)
Trust in religious organizations	0.05	1.05	(0.84, 1.30)		0.04	1.04	(0.83, 1.30)		0.01	1.01	(0.80, 1.28)
Knowledge					0.73	2.07*	(1.63, 2.63)		0.82	2.26*	(1.75, 2.92)
Expert primary source									0.19	1.20	(0.62, 2.33)
Internet primary source									-0.21	0.81	(0.41, 1.59)
Government primary source									0.10	1.10	(0.72, 1.67)

*p < 0.001; Covariates: gender, race (white vs. others), political ideology (liberal vs. others), higher education level, marital status (single vs. others), general health (good vs. poor), religious ideology (atheist vs. others), age, and insurance status (insured vs. not insured). Referent categories for information source were all other sources selected as the first source of vaccine information

Expert primary source: Doctor/health care worker as the first source of information (vs. other choices [ranks 2–13])

Internet primary source: Internet as the first source of information (vs. other choices [ranks 2–13])

#### Supplementary results

Models 5, 6, and 7 in Table 5 demonstrate an individual association between trust variables and vaccine confidence, between vaccine knowledge in addition to existing trust variables and vaccine confidence, and between information source variables in addition to existing vaccine knowledge and trust variables and vaccine confidence, respectively. First, trust in doctors (OR = 2.06, *p* < 0.001, 95%CI 1.56, 2.69) and trust in government health agencies (OR = 2.68, *p* < 0.001, 95% CI 2.06, 3.49) had higher odds of vaccine confidence (see Model 5 in Table 5). These results indicate that as trust in the specified entities increase, vaccine confidence increases as well.

.

Government primary source: Government websites as the first Internet source for information (vs. other choices [ranks 2–13])

When vaccine knowledge was added to the model (see Model 6 in Table 5), vaccine knowledge (OR = 2.07, *p* < 0.001, 95%CI 1.63, 2.63) had significantly increased odds relating to vaccine confidence. Trust in doctors (OR = 1.90, *p* < 0.001, 95%CI 1.43, 2.52) and trust in government health agencies (OR = 2.69, *p* < 0.001, 95%CI 2.04, 3.53) retained their significant associations with vaccine confidence. When comparing Model 5 to Model 6, trust in doctors showed decreases in odds ratio by 7.28%, while the odds ratio for trust in government health agencies increased by 1.49%. The observed decreases indicate that trust in doctors may conceptually overlap with vaccine knowledge.

When three information source variables were added to the model (see Model 7 in Table 5), none were significantly associated with vaccine confidence. When comparing Model 6 to Model 7, trust in doctors and government health agencies showed increases in odds ratios by 0.53% and 1.12%, respectively. Vaccine knowledge also showed an increase in odds ratio by 9.18%. The observed increase in odds ratios among two trust variables and vaccine knowledge further suggest that trust and vaccine knowledge might be established through the use of governmental sources for vaccine information. Higher trust in doctors and government health agencies and greater vaccine knowledge cement vaccine confidence, and vice versa.

## Discussion

### Summary

The objectives of this research were to examine the association between vaccine confidence and trust, knowledge, and information sources, while accounting for covariates and to compare the relative strength of vaccine confidence sources. Trust was the strongest factor contributing to vaccine confidence, and although lesser, knowledge also helped contribute.

The findings indicate that independently, trust (in doctors, in government, and in charitable organizations), vaccine knowledge, and information sources contribute to vaccine confidence even after socioeconomic and demographic characteristics were controlled. When trust, vaccine knowledge, and information sources were collectively compared, trust was the most significant contributor, particularly trust in government health agencies. This supports previous studies in that trust is one of the most important factors in predicting and establishing vaccine confidence [38, 39].

The observed decreases in odds ratios for trust in doctors after the introduction of vaccine knowledge variable appear to indicate that vaccine knowledge and trust variables might be conceptually similar. In fact, several studies have noted the strong association between cognitive trust and knowledge, as the fundamental basis of developing cognitive trust in a source is its high level of knowledge and expertise [40][41]. The potential conceptual overlap between participants’ vaccine knowledge and their cognitive trust in doctors may explain decrease in beta values and odds ratios of trust in doctors across different models.

Individuals’ considering the government the primary source of information was significantly associated with vaccine confidence when only information sources are considered in the logistic regression model (i.e., Model 3). However, none of the information source variables were significantly associated with vaccine confidence when other confidence sources were included in the logistic regression model (i.e., Model 4). This may be due to the fact that the strongest predictor of vaccine confidence, trust in government health agencies, conceptually overlaps with the likelihood of seeking government as the primary information source. Moreover, although information sources themselves may not explain confidence, it could serve as a gateway to build reliable vaccine knowledge and ultimately, trust. Research has noted similar findings in that while exposure to information or information searching behaviors may not have a direct effect on vaccine confidence, it is essential in the establishment of trust [42]. Further, several studies have utilized the social cognitive (learning) theory demonstrated that communication and information are essential components to health beliefs and attitudes [43, 44].

### Implications

When the relationship between vaccine confidence and all independent variables was assessed, trust in government health agencies and vaccine knowledge consistently remained as strongest predictors of vaccine confidence. This may indicate that trust variables have dimensions contributing to vaccine confidence that information sources may not explain. This may reflect the fact that trust is complex, with cognitive, affective, and behavioral trust all contributing overall to trust in general. Future researchers are encouraged to carefully evaluate and measure cognitive, affective, and behavioral trust as well as overall trust and examine the relationships among multiple dimensions of trust, vaccine knowledge, information sources, and vaccine confidence.

Digital and social media have been considered the culprit in the emergence of “infodemic” [45] and vaccine hesitancy as well as the global erosion of trust both in academic literatures [46] and public discourses [47][48]. The findings of this study imply that such reflects a simplistic view. The findings of this study provide a glimpse of hope in that, individuals could build correct vaccine knowledge and strengthen trust in doctors and government health agencies, if they were to be exposed to well-executed vaccine promotion campaigns and education via government sources. This involves a systems approach where academics and healthcare providers make collective efforts to better translate scientific findings into consumer digital content; governmental organizations and technology companies collaboratively work together to develop and incentivize algorithms where verified sources of vaccine information appears as the top search results; and science/medical experts and educators continue to make an effort to increase digital information literacy among general public to discern information [49, 50].

Given the complex nature of trust and other factors influencing individuals’ health decisions, vaccination campaigns should incorporate trust, knowledge, and media consumption to re-build confidence especially in rural communities. One of the examples is to use trust messengers. In fact, several studies have attempted to incorporate trust messengers in an attempt to provide factual information from stakeholders in rural communities in liberal states, and they have been very well received [51, 52].

Our results highlighting the importance of trust as a key contributor toward building vaccine confidence provide practical implications for public health professionals. Given the importance of knowledge and trust in vaccine confidence, successful public health efforts promoting vaccinations are encouraged to include informative content and consistent and transparent efforts to continue to build the public’s trust in vaccinations. There is a need for collaboration between local, state, and federal public health agencies, major search engines, and government internet media platforms. Increased prominence of Internet sources providing accurate information, such as government or educational websites, may prevent undermining vaccine confidence to a greater extent. Moreover, improving virtual accessibility to traditionally trusted sources, such as books, brochures, and health care providers, may contribute to restoring vaccine confidence.

The findings of this study are specifically useful in informing rural vaccine interventions. Integrating university and community stakeholders, such as student health services on college campuses, local healthcare systems, and local and state health departments, into implementation efforts is highly recommended. Not only do they represent significant trust entities, namely, doctors and government health agencies, respectively, but they are also more likely to have a strong digital and social media presence, compared to brand-new interventions and promotions. In particular, partnering with existing local and state health departments’ websites and social media platforms to deliver interventions and interact with audience would be advantageous. These efforts would contribute to increasing trust, while increasing vaccine knowledge and providing applicable resources, to ultimately increase vaccine confidence.

### Limitations

This study has several limitations. The findings of this study may not be applicable to other regions and states across the U.S. The exclusion of remote employees and online students limit the generalizability of this study’s findings. Given the prevalence of vaccine hesitancy worldwide, future research would benefit from the inclusion of remote employees and online students who tend to bring diverse cultural and lived experiences.

The findings of this study were from a cross-sectional survey, which does not establish causality. It indicates that the interpretation of study findings of study avoids using any causality claims. It also suggests that increase in trust and knowledge as well as increase in vaccine confidence is a circular process where greater trust and knowledge contributes to increasing in vaccine confidence, and greater vaccine confidence also contributes to further strengthening trust and knowledge.

Further, the recruitment methods used in this study may introduce selection bias. Considering the polarization of vaccines in America, social-desirability bias may have influenced participant response. Additionally, the findings of this study do not suggest how to build confidence, but rather suggestions for allocating resources toward information sources and communication strategies. It is important to acknowledge that socioeconomic, personal risk perception, cultural and historical contexts, and community influence contribute to vaccine confidence.

## Conclusion

This research highlighted sources contributing to vaccine confidence in North Dakota, one of the most conservative states in the U.S. where it faces a significantly lower rate of vaccine uptakes. The finding showed a significant association among trust in doctors, government health agencies, vaccine knowledge, and vaccine confidence. Among them, trust in government health agencies and vaccine knowledge were most strongly associated with vaccine confidence. In addition, individuals’ reliance on government sources as the primary source of vaccine information provides meaning implications for building vaccine confidence. This encourages multiple stakeholders in public health and digital technology to develop mechanisms by which verified scientific information is prioritized for individuals seeking vaccine information online. Local, state, and federal public health agencies, as well as search engines and social media platforms should ensure prominent and accessible virtual sources that contain accurate information. Further, the results suggest that interventions for vaccine promotion, especially in rural areas and small college towns, are encouraged to collaborate university and community stakeholders not only to represent trustworthy sources, but also to utilize their existing digital and social media presence. Strategic resource allocation and campus and community partnership would build trust and knowledge via effective communication channels, ultimately contributing to vaccine confidence.

## Data Availability

The datasets generated and/or analyzed during the current study are not publicly available due to ongoing analysis but are available from the corresponding author on reasonable request.

## Abbreviations

CI: Confidence intervals
CDC: Centers for Disease Control and Prevention
OR: Odds ratio
SPSS: Statistical Package for the Social Sciences
